# Gastric microbiota dysbiosis and *Helicobacter pylori* infection

**DOI:** 10.3389/fmicb.2023.1153269

**Published:** 2023-03-30

**Authors:** Ling Zhang, Ming Zhao, Xiangsheng Fu

**Affiliations:** Department of Gastroenterology, Clinical Medical College, The First Affiliated Hospital of Chengdu Medical College, Chengdu, Sichuan, China

**Keywords:** gastric microecology, gastric diseases, *H. pylori* eradication, bacterial interaction, microbiota transplant

## Abstract

*Helicobacter pylori* (*H. pylori*) infection is one of the most common causes of gastric disease. The persistent increase in antibiotic resistance worldwide has made *H. pylori* eradication challenging for clinicians. The stomach is unsterile and characterized by a unique niche. Communication among microorganisms in the stomach results in diverse microbial fitness, population dynamics, and functional capacities, which may be positive, negative, or neutral. Here, we review gastric microecology, its imbalance, and gastric diseases. Moreover, we summarize the relationship between *H. pylori* and gastric microecology, including non-*H. pylori* bacteria, fungi, and viruses and the possibility of facilitating *H. pylori* eradication by gastric microecology modulation, including probiotics, prebiotics, postbiotics, synbiotics, and microbiota transplantation.

## 1. Introduction

The stomach was historically assumed to be a sterile organ due to its acidic pH and peristaltic movement. However, this assumption was corrected with the discovery of *Helicobacter pylori* (*H. pylori*), which is a gram-negative bacterium that mainly colonizes the human stomach (Marshall and Warren, 1984). Although the majority of *H. pylori*-infected individuals remain asymptomatic, chronic infections are strongly correlated with chronic gastritis, peptic ulcer diseases, gastric cancer (GC), and mucosa-associated lymphoid tissue lymphoma (Peek and Blaser, 2002; Tsai and Hsu, 2010; Wang et al., 2014). *H. pylori* infections are also associated with extragastrointestinal (GI) diseases, such as autoimmune diseases, idiopathic thrombocytopenic purpura, iron-deficiency anemia, and cardiovascular and cerebrovascular diseases (Santos et al., 2020). *H. pylori* colonization and pathogenesis are influenced by multiple factors, including urease, adhesins, outer membrane proteins, neutrophil-activating protein A, cytotoxin-associated gene A (CagA), vacuolar cytotoxin A (VacA), and the type IV secretion system (T4SS) (Kronsteiner et al., 2016). With the success of eradication regimens and improvements in sanitation, the prevalence of *H. pylori* is decreasing worldwide, especially in developed countries (Burucoa and Axon, 2017; Hooi et al., 2017). However, a substantial drop in *H. pylori* treatment efficacy has been noted due to increasing antibiotic resistance, making the development of new treatment strategies crucial (Megraud et al., 2021; Tshibangu-Kabamba and Yamaoka, 2021).

The microbiome is a complex microbial community comprising bacteria, fungi, and viruses residing in distinct human body habitats with strong niche specialization (Human Microbiome Project Consortium, 2012). Molecular technologies, such as whole genome 16S ribosomal RNA (rRNA) sequencing and metagenomics, transcriptomics, proteomics, and metabolomics studies (Barra et al., 2021), have provided a better understanding of the gastric microenvironment. The gastric niche is modulated by various factors, including diet, antibiotics, histamine type 2 (H_2_) antagonists, proton pump inhibitors (PPIs), probiotics, and *H. pylori* infection (Sterbini et al., 2016; Brawner et al., 2017). *H. pylori* and other microbial communities have complex interactions within the unique gastric microecological environment. This review focuses on the relationship between *H. pylori* and other microorganisms.

## 2. Gastric microecology formation

**FIGURE 1:**
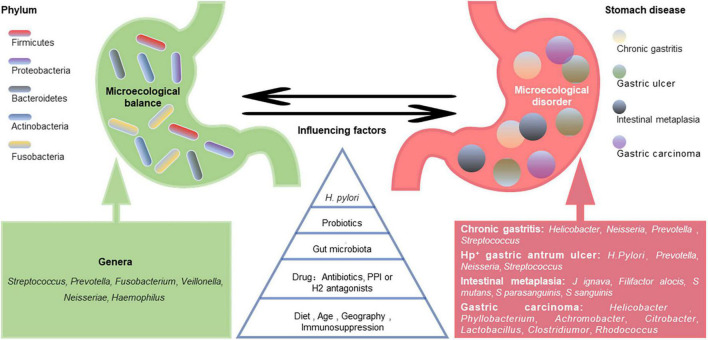
Gastric microecological imbalance and gastric diseases. Despite the differences among individuals, there are five dominant bacterial phyla in the healthy stomach, and their common dominant bacterial genera are summarized (green). The gastric microbiota is dynamically balanced and affected by many factors, such as *Helicobacter pylori* infection, probiotics, gut microbiota, drugs, diet, and age. Although the causal relationship between them is unclear, gastric microecological imbalances are associated with various gastric diseases (red), and some microorganism-related disorders are listed.

A healthy stomach is colonized by diverse microbiota. Large differences in gastric microbiota composition among individuals have been observed. Bacterial communities in healthy stomachs have not been extensively characterized. However, studies have found that Firmicutes, Proteobacteria, Bacteroidetes, Actinobacteria, and Fusobacteria are the most prominent phyla in gastric mucosa (Bik et al., 2006), and *Streptococcus*, *Prevotella*, *Fusobacterium*, *Veillonella*, *Neisseria*, and *Haemophilus* are the most prevalent genera (Bik et al., 2006; Li et al., 2009; Delgado et al., 2013; Engstrand and Lindberg, 2013; Ndegwa et al., 2020; Figure 1). Compared with those in the gastric mucosa, *H. pylori* and Proteobacteria levels were relatively decreased in gastric fluid, while Actinobacteria, Bacteroidetes, and Firmicutes were increased (Sung et al., 2016). It should be noted that gastric fluid samples showed higher diversity than gastric mucosa samples. However, bacteria in gastric juice may be transient since the stomach is exposed to bacterial influx from the oral cavity and reflux *via* the duodenum (Nardone and Compare, 2015).

The gastric microbiota composition is highly dynamic, as it changes with *H. pylori* infection, antibiotic exposure, probiotic consumption, PPI or H2 antagonist use, dietary habits, age, vitamin supplementation (especially D3), immunosuppression, and potentially geography and gut microbiota (Espinoza et al., 2018; Figure 1). A long-term follow-up study of *H. pylori*-negative individuals without atrophic gastritis and intestinal metaplasia (IM) showed that microbial diversity and Firmicutes and Fusobacteria abundances decreased while Proteobacteria phylum abundance increased with age (Shin et al., 2020). However, another study showed that age and sex did not significantly affect the bacterial composition of the stomach (Li et al., 2017). Some ethnicities have specific microbiota profiles. For example, *Micrococcus luteus* and *Sphingomonas yabuuchiae* were significantly associated with the Timor and Papuan ethnicities, respectively, (Miftahussurur et al., 2020). A cross-sectional study focusing on minority ethnic groups in Vietnam showed that the prevalence of *H. pylori* infection was significantly higher in Nung living in Daklak than in Lao Cai (Binh et al., 2018). Das et al. (2017) found that while the microflora of samples from the USA and Colombia were similar, those from India and China appeared closer. The differences in gastric flora among individuals of different ethnicities or regions may be partly related to their dietary habits. China is a country with high salt intake, which is twice the value recommended by the WHO (Zhang et al., 2020). A high-salt diet primarily changes the composition of the gastric microbiota by reducing the relative abundance of Bacteroidetes and Proteobacteria at the phylum level and decreasing the relative abundances of Unclassified_S24-7 and *Lactobacillus* at the genus level (Li Y. et al., 2020).

The establishment and stability of the gastric microecology is attributed to the mucus barrier, biological barrier and immune system. The mucus layer establishes a pH gradient, with a pH of 1–2 in the gastric lumen and 6–7 at the mucosal surface (Bhaskar et al., 1992). The gastric juice-derived bacteria and their DNA develop a barrier to weaken most bacterial colonization, while the bacteria adhering to the mucosa create a more hospitable environment for colonization (Hunt et al., 2015). The gastric innate and adaptive immune responses maintain microbial balance through the immune homeostasis mechanism. Recent evidence has shown that the reciprocal interaction between the type 2 innate lymphoid cells (ILC2s) and commensal microbiota of the stomach maintains the homeostasis of the microbial environment (Satoh-Takayama et al., 2020).

## 3. Gastric microecological imbalance and gastric diseases

Altered gastric microbiota composition and function are considered gastric ecological disorders and can be induced by various environmental factors. Microecological disorders can cause gastric immune dysfunction, decrease dominant bacteria, and increase the abundance and virulence of pathogenic microorganisms, leading to pathogenic bacterial invasion and related diseases (Figure 1). Compared with *H. pylori*-infected germ-free (GF) INS-GAS mice, *H. pylori*-infected specific pathogen-free (SPF) INS-GAS mice developed more severe gastric lesions and earlier GI intraepithelial neoplasia (Lofgren et al., 2011). This finding supports the view that the gastric microbiota may contribute to gastric disease following *H. pylori* infection.

Hypochlorhydria patients have many urease-positive bacteria other than *H. pylori*, such as *Actinomyces*, *Corynebacterium*, *Haemophilus*, *Streptococcus*, and *Staphylococcus* (Brandi et al., 2006). *Lactobacillus* and *Enterococcus* are commensal bacteria in healthy stomachs, with abundances up to 30 and 51%, respectively. However, exceeding these limits is thought to be a risk for GC (Gantuya et al., 2020).

The predominant bacterial phyla in *H. pylori*-positive gastric antrum ulcers were Proteobacteria, Bacteroidetes, and Firmicutes. *H. pylori* was dominant at the genus level, followed by *Prevotella*, *Neisseria*, and *Streptococcus* (Chen et al., 2018). *Johnsonella ignava* and *Filifactor alocis* were enriched in patients with gastric IM compared with healthy control individuals, and *Streptococcus mutans*, *Streptococcus parasanguinis*, and *Streptococcus sanguinis* were depleted. The sugar degradation pathways of gut microbiota were also depleted in IM patients, while the lipopolysaccharide and ubiquinol biosynthesis pathways were more abundant (Wu et al., 2022).

Ferreira et al. (2018) found that *Helicobacter*, *Neisseria*, *Prevotella*, and *Streptococcus* were more abundant in patients with chronic gastritis. There was a significant decrease in *Helicobacter* in gastric carcinoma, while the *Phyllobacterium*, *Achromobacter*, *Citrobacter*, *Lactobacillus*, *Clostridium*, and *Rhodococcus* genera were more abundant (Ferreira et al., 2018). Changes in bacterial diversity during GC progression are inconsistent across studies. Some studies have shown a progressive decline in microbial diversity from gastritis to cancer (Aviles-Jimenez et al., 2014), while others have found increases in bacterial diversity during this process (Eun et al., 2014). These differences potentially reflect the different microbiota characterization platforms and study populations used across studies. The pathogenic mechanisms of gastric microorganisms, including *H. pylori*, may include inducing the inflammatory response, influencing the function of immune cells in the tumor microenvironment, and producing harmful metabolites, such as N-nitroso compounds (Li and Yu, 2020).

Gastric microecology is also affected by drug use. For example, vancomycin reduced the abundance of Actinobacteria and Bacteroidetes phyla (Satoh-Takayama et al., 2020). In addition, PPI-treated patients had more *Streptococcus* than patients with normal gastric mucosa (Parsons et al., 2017) and dyspeptic patients without PPI treatment (Sterbini et al., 2016).

## 4. Gastric microecology and *H. pylori* infection

### 4.1. *H. pylori* infection affects gastric microecology

*Helicobacter pylori* infection has been reported to modulate gastric microbe diversity (Das et al., 2017). *H. pylori*^+^/CagA^+^ samples showed lower gastric microflora diversity and *Roseburia* abundance but higher *Helicobacter* and *Haemophilus* genera abundances than healthy or *H. pylori*^+^/CagA^–^ samples (Zhao et al., 2019). The relative abundances of phyla, including Actinobacteria, Bacteroidetes, Firmicutes, Fusobacteria, Gemmatimonadetes, and Verrucomicrobia, were significantly decreased in *H. pylori*^+^ children compared to *H. pylori*^–^ children. Nine genera differed in abundance between *H. pylori*^+^ and *H. pylori*^–^ children, including *Helicobacter*, *Achromobacter*, *Devosia*, *Halomonas*, *Mycobacterium*, *Pseudomonas*, *Serratia*, *Sphingopyxis*, and *Stenotrophomonas* (Zheng et al., 2021).

*Helicobacter pylori* acts to produce urease, which transforms urea into carbon dioxide and ammonia to neutralize the acidic environment of the stomach to facilitate its colonization (Weeks et al., 2000). Acute infection can lead to hypochlorhydria, while chronic infection at different anatomical sites can result in hypo- or hyperchlorhydria. Changes in acid secretion caused by *H. pylori* may allow ingested microorganisms to survive transit through the stomach (Smolka and Schubert, 2017). There has been a hypothesis that while reduced gastric pH during acute *H. pylori* infection leads to colonization, elevated gastric pH during chronic *H. pylori* infection leads to a microbial bloom that further inhibits *H. pylori* growth (Das et al., 2017).

### 4.2. Gastric microecology affects the host response to *H. pylori*

#### 4.2.1. Bacteria

The varied host responses to *H. pylori* infection may be attributed to gastric microbe diversity and abundance (Table 1). A family-level analysis of bacterial abundance showed apparent differences between the C57BL/6 mice from Jackson Laboratory (Jax) and the C57BL/6 mice from Taconic Sciences (Tac), accompanied by different responses to *H. pylori* infection. *H. pylori*-infected Jax mice had higher *H. pylori* colonization levels and gastric *IL-1*β and *IL-17A* transcription, while *H. pylori*-infected Tac mice had more severe metaplasia of the gastric mucosa and a stronger Th1-associated IgG2c response to *H. pylori*. In addition, the energy metabolism, amino acid, and secondary metabolite biosynthesis pathways were upregulated in the microbiota that resided in the stomach of Jax mice. In contrast, lipid, cofactor, vitamin metabolism and xenobiotic biodegradation were elevated in the stomach of Tac mice. The difference in gastric bacterial community structures could potentially regulate distinct pathways, which could affect stomach physiology and lead to different *H. pylori* infection responses (Ge et al., 2018).

**TABLE 1:** The gastric microecology affects the host response to *H. pylori*.

Research object	Subject Country	Sample Type	Major Findings	References
**Bacteria**				
Mice	USA	Gastric tissue	Mice with different microbiota can produce different host immune responses and pathological changes induced by *H. pylori*.	Ge et al., 2018
Mice	USA	Gastric tissue	Mice coinfected with *H. pylori* and *S. salivarius* had more severe gastritis, while coinfection with *S. epidermidis* and *H. pylori* could reduce the pro-inflammatory response.	Shen et al., 2022
Children	China	Gastric tissue	The gastric microbiota of *H. pylori*-infected children might produce short-chain fatty acids and small molecules that modulate mucosal Treg responses to favor the persistence of bacteria.	Zheng et al., 2021
Mice	Japan	Gastric tissue	Gastric ILC2, regulated by the commensal microbiota, were important in clearance of infectious *H. pylori* by inducing IgA-producing plasma cells.	Satoh-Takayama et al., 2020
**Fungus**				
Gastric yeast	Iran	_	Yeast vacuole can serve as a specialized niche for *H. pylori* to enhance bacterial survival.	Siavoshi et al., 2019
**Virus**				
The gastric cancer EBV-negative cell line (AGS)	India	_	Coinfection with EBV and *H. pylori* improved the expression of EBV latent genes and *H. pylori*-associated genes.	Kashyap et al., 2021
Patients and human gastric cancer cells	Japan	Gastric tissue	Host SHP1 could be downregulated by EBV to enhance *H. pylori* CagA activity.	Saju et al., 2016

*Helicobacter pylori* infection modulates the host immune system in profound ways, including suppression of T helper 17 (Th17) cells and induction of regulatory T (Treg) cells (Lehours and Ferrero, 2019). Commensal gastric microbes or their metabolites influence the capability of *H. pylori* to colonize the stomach and its pathogenic and carcinogenic potential by modulating host immune responses (Espinoza et al., 2018). The presence of non-*H. pylori* bacteria might persistently act as an antigenic stimulus or establish a partnership with *H. pylori* to enhance subsequent inflammation (Rook et al., 2017). Stomach-derived urease-positive *Staphylococcus epidermidis* and *Streptococcus salivarius* were independently inoculated into GF INS-GAS mice with *H. pylori*. The gastric pathology of the latter was significantly higher than in mice only infected with *H. pylori*. In contrast, the proinflammatory cytokine responses (*IL-1*β, *IL-22*, IFN-γ, and TNF-α) of the former were significantly lower than those in mice only infected with *H. pylori* (Shen et al., 2022). Studies have found that the *IL-17A* to *FOXP3* mRNA ratio was inversely correlated with *H. pylori* abundance in infected children. Moreover, gastric microbial communities significantly upregulated their alpha-linolenic acid and arachidonic acid metabolism. Therefore, Zheng et al. (2021) hypothesized that the balance between Treg and Th17 cells might be biased toward Treg cells, which is beneficial to bacterial persistence, and the gastric microbiota might generate short-chain fatty acids (SCFAs) and small molecules to modulate mucosal Treg responses in *H. pylori*-infected children. In addition, Satoh-Takayama et al. (2020) showed that ILC2s, regulated by local commensal communities through *IL-7* and *IL-33* induction, are the predominant ILC subset in the stomach and protect against *H. pylori* infection through B-cell activation and IgA production.

#### 4.2.2. Fungus

*Candida albicans* is one of the most common fungi in the human body. In Karczewska et al. (2009) reported the coexistence of *H. pylori* with *Candida* in patients with gastric ulcers, suggesting their synergy in disease pathogenesis. *H. pylori* is a facultative intracellular bacterium that may protect itself against environmental stress by entering *C. albicans* cells, allowing the invading *H. pylori* to be transmitted to subsequent *C. albicans* generations (Siavoshi and Saniee, 2014; Siavoshi et al., 2019). Siavoshi et al. (2019) found that the yeast vacuole served as a sophisticated niche for *H. pylori*, with sequestration inside the vacuole potentially enhancing bacterial survival. It should be noted that the proportion of yeast cells harboring bacteria in an acidic environment was nearly twice that in a neutral environment. However, when the pH is < 4, the number of bacteria-invaded yeast cells decreases sharply (Sanchez-Alonzo et al., 2020). In addition, temperature, anaerobic environment, nutritional condition, and drugs (e.g., amphotericin B) might affect the entry or exit of *H. pylori* in *Candida* cells (Sanchez-Alonzo et al., 2021a,c, 2022; Tavakolian et al., 2018). *H. pylori* has been reported to invade vaginal yeast cells, causing vertical transmission during birth (Sanchez-Alonzo et al., 2021b). In addition, *C. albicans* harboring *H. pylori* is also abundant in honeybees, honey, flowers, and natural fruits (Siavoshi et al., 2018). These results suggest that we can reduce or prevent *H. pylori* transmission through fungal interventions.

#### 4.2.3. Virus

*Helicobacter pylori* and Epstein–Barr virus (EBV) have been reported to cooperate to induce more severe gastritis than each alone. Their combined infection promotes host expression of the oncogenic protein gankyrin and the oncogenic properties of human gastric adenocarcinoma cells (AGS) (Cárdenas-Mondragón et al., 2013; Kashyap et al., 2021). Higher expression of latent EBV nuclear antigen 1 and 3C (*ebna1* and *ebna3c*) genes was observed at 12 and 24 h in samples coinfected with *H. pylori* and EBV compared with EBV alone. Similarly, the expression levels of the *H. pylori*-associated genes *16S rRNA*, ***C**agA*, and blood-group antigen-binding adhesin (*babA*) were higher in coinfected cells than in cells infected with *H. pylori* alone (Kashyap et al., 2021). Pandey et al. (2018) showed that the CagA protein of *H. pylori* promoted EBV-mediated proliferation of coinfected cells. EBV enhanced *H. pylori* CagA activity by downregulating one of its host antagonists, Src homology region 2 domain-containing phosphatase-1 (Saju et al., 2016).

## 5. *H. pylori* eradication and gastric microecology

Radical *H. pylori* treatment regimens involve PPI triple therapy, bismuth-containing quadruple therapy, modified regimens (modified bismuth-containing quadruple regimen, high-dose dual therapy, and vonoprazan-containing regimens), concomitant therapy, hybrid therapy, and sequential therapy (Liu et al., 2021). While the eradication of *H. pylori* affects gastric microbial composition and function, whether *H. pylori* eradication restores the gastric microbiota to an uninfected status remains controversial (Guo et al., 2022). Predictable factors affecting gastric microecological recovery after *H. pylori* eradication might include atrophy/metaplasia in the basal state, higher neutrophil infiltration at the corpus, lower pepsinogen (PG) I/II ratio, and higher relative *Acinetobacter* abundance (Shin et al., 2020). A recent meta-analysis showed that the gastric microbial composition changed significantly after quadruple or triple therapy, with relative *H. pylori*-related taxa abundance (Proteobacteria phylum and *Helicobacter* genus) decreasing to different degrees. In contrast, typically dominant gastric commensals (e.g., Firmicutes, *Bacteroides*, and Actinobacteria) were enriched after *H. pylori* eradication (Guo et al., 2022). Studies exploring changes in gastric microbiota functions after *H. pylori* eradication found that bacterial reproduction-related pathways, such as flagellar assembly, chemotaxis, and nucleotide-binding oligomerization domain (NOD)-like receptor signaling, were downregulated in gastric microbiota. In contrast, normal gastric function-related pathways, such as gastric acid secretion, protein digestion and absorption, and amino acid metabolism, were upregulated (He et al., 2019; Guo et al., 2020; Sung et al., 2020).

Antibiotic treatment leads to the widespread destruction of bacterial community structures. Human microbiome reconstitution after antibiotic treatment is usually slow and incomplete (Suez et al., 2018). With increasing antibiotic resistance, guidelines recommend bismuth quadruple therapy as the first-line treatment (Fallone et al., 2019). There is evidence that the effectiveness of bismuth-containing quadruple *H. pylori* eradication therapy depends on gastric microbiota, as high *H. pylori* eradication rates are associated with *Lactobacillus, Rhodococcus*, and *Sphingomonas* (Niu et al., 2021).

## 6. *H. pylori* eradication *via* gastric microecology modulation

### 6.1. Probiotics

Probiotics are living microorganisms that benefit the host when administered in adequate amounts (Hill et al., 2014). They have been shown to reduce *H. pylori*-induced gastric pathology in mice, with reduced inflammatory infiltration and precancerous lesion incidence (He et al., 2022). They also enhance *H. pylori* eradication rates and reduce side effects in humans (Zhang et al., 2015; McFarland et al., 2016; Fang et al., 2019; Viazis et al., 2022). Yuan et al. (2021) explored the effect of probiotic-supplemented quadruple therapy on gastric microecology. *Bifidobacterium* and *Lactobacillus* were enriched in the gastric mucosa and juice, respectively, of the probiotic-supplemented group compared to the quadruple therapy group. In contrast, the levels of potentially pathogenic bacteria, including *Fusobacterium* and *Campylobacter*, were decreased. Microbial diversity was closer to that of *H. pylori*-negative subjects after probiotic-supplemented eradication treatment (Yuan et al., 2021).

Currently, probiotics with potential activity against *H. pylori* infection belong to the Firmicutes (*Enterococcus* and *Lactobacillus*) and Actinobacteria (*Bifidobacterium* genus) phyla and *Saccharomyces boulardii* (Keikha and Karbalaei, 2021). The most commonly proposed mechanisms underlying the probiotic effects include inhibiting pathogens, producing useful metabolites or enzymes, and modulating immunity. In addition, quorum sensing is considered to be one of the mechanisms of probiotics regulating the restoration of the gastric microbiota. Probiotics may exert beneficial effects through one or more of these pathways (Table 2).

**TABLE 2:** The possible mechanisms of probiotics against *H. pylori*.

Probiotic species	Subject country/ district of origin	Mode of action	Major findings	References
**Pathogen inhibition**				
*Lactobacillus reuteri*	Japan	Sulfatide-binding protein	Inhibiting the binding of *H. pylori* to the glycolipid receptors competitively.	Mukai et al., 2002
*Saccharomyces boulardii*	Türkiye	Neuraminidase activity	Removing surface α (2-3)-linked sialic acid.	Sakarya and Gunay, 2014
*Lactobacillus rhamnosus* JB3	Taiwan	Bacteria and the cell-free supernatant	Reducing the expression of *H. pylori* virulence gene. Suppressing Lewis (Le)^x^ antigen, TLR4, and the α5β1 integrin expressions in AGS cells.	Do et al., 2021a,b
**Bacterial metabolites**				
Lactic acid bacteria	Canada	The cell-free supernatant	Affecting flagella-mediated motility, inhibiting *H. pylori* growth, urease activity, and the secretion of *IL-8*.	Whiteside et al., 2021
Lactic acid bacteria	Republic of Korea	Bacteriocin	Expression of anti-*H. pylori* activity.	Kim et al., 2003
*Lactobacillus delbrueckii* subsp. *bulgaricus* strains	Bulgaria	The cell-free supernatant	Producing bacteriocin-like inhibitory substances.	Boyanova et al., 2017
**Modulation immunity**				
*Lactobacillus salivarius*; *Lactobacillus rhamnosus*	China	Probiotic combination	Decreased expression levels of genes involved in pro-inflammatory pathways.	He et al., 2022
*Lactobacillus* spp.	Ireland; Taiwan; Thailand	Bacteria	Inhibiting the secretion of *IL-8* by *H. pylori* infected AGS cells in different ways.	Ryan et al., 2009; Yang et al., 2012; Thiraworawong et al., 2014
*Lactobacillus fermentum* P2, *L. casei* L21, *L. rhamnosus* JB3	Taiwan	Single probiotic or combination	Decreasing IFN-γ, *IL-1*β, *H. pylori* specific IgA, IgM levels.	Lin et al., 2020
**Communication between microorganisms—quorum sensing**				
*Lactobacillus rhamnosus* JB3	Taiwan	The cell-free supernatant	Secreting an unknown bioactive substance to act as an antagonist of AI-2.	Do et al., 2021a

#### 6.1.1. Pathogen inhibition

*Lactobacillus reuteri* inhibits *H. pylori* attachment by competitively binding to gastric epithelial gangliotetraosylceramide (asialo-GM1) and sulfatide (Mukai et al., 2002). *S. boulardii* produces neuraminidase selective for α (2–3)-linked sialic acid to remove *H. pylori* adhesin ligands, inhibiting *H. pylori* adherence to host cells (Sakarya and Gunay, 2014). Do et al. (2021a,b) used a cell model to show that *Lactobacillus rhamnosus* JB3 (LR-JB3) reduced *H. pylori* VacA, sialic acid-binding adhesin (SabA), and fucosyltransferases (FucT) and decreased Lewis (Le)*^x^* antigen, toll-like receptor 4 (TLR4) and α5β1 integrin expression in AGS cells. Therefore, it further suppressed lipid raft clustering and attenuated Lewis antigen-dependent adherence, T4SS-mediated cell contact, and lipid-raft-mediated VacA entry into host cells (Do et al., 2021a,b).

#### 6.1.2. Bacterial metabolites

Probiotic-derived metabolites have been extensively studied in *H. pylori* eradication. Cell-free lactic acid bacterial culture supernatants reduced *H. pylori* growth, urease activity, flagella-mediated motility, and *H. pylori*-induced host *IL-8* secretion (Whiteside et al., 2021). The probiotics produced bacteriocins, such as *Lacticins* A164 and BH5, that could antagonize the proliferation of *H. pylori* (Kim et al., 2003). *Lactobacillus brevis* BK11 and *Enterococcus faecalis* BK61 reduced *H. pylori* urease activity and adhesion to cultured human gastric adenocarcinoma epithelial cells (Lim, 2015). In addition, Boyanova et al. (2017) found that bacteriocin-like inhibitory substances from *Lactobacillus delbrueckii* subsp. *bulgaricus* strains could kill antibiotic-susceptible and antibiotic-resistant *H. pylori*.

Probiotics can reshape the gastric microbiota structure. Probiotic administration enhanced the proportion of beneficial SCFA-producing bacteria, including *Bacteroides*, *Alloprevotella*, *Oscellibacter*, in the stomachs of *H. pylori*-infected mice (He et al., 2022). Sodium butyrate, one of the representative SCFAs, not only inhibited *H. pylori* growth and *CagA* and *VacA* expression, but also inhibited the host NF-κB pathway by reducing toll-like receptor expression in host cells to decrease TNF-α and *IL-8* production (Huang et al., 2021). However, another bacterial metabolite, trimethylamine *N*-oxide (TMAO), increased *H. pylori* viability and virulence and exacerbated *H. pylori*-induced inflammation (Wu et al., 2020). The synergistic effects of *H. pylori* and TMAO enhanced inflammation-related gene expression, including *IL-6*, C-X-C motif chemokine ligands 1 and 2 (CXCL1, CXCL2), FOS, and complement C3 in the gastric epithelium (Wu et al., 2017). Trimethylamine (TMA) is the TMAO precursor. It is mainly produced by Firmicutes (e.g., *Staphylococcus*) and relatively rare in Bacteroidetes (Fennema et al., 2016). Overall, probiotics may increase the proportion of beneficial metabolite-producing bacteria and/or reduce the proportion of harmful metabolite-producing bacteria.

#### 6.1.3. Immunity modulation

Increasing evidence has suggested the role of probiotics in immune modulation in *H. pylori*-infected animal models. The combined administration of probiotic *Lactobacillus salivarius* and *Lactobacillus rhamnosus* attenuated inflammatory pathways, such as NF-κB, *IL-17*, and TNF-α, in *H. pylori*-infected mice (He et al., 2022). Several *Lactobacillus* spp. isolates have been reported to reduce *IL-8* secretion by *H. pylori*-infected AGS cells. These include decreasing Cag secretory system function (Ryan et al., 2009), inactivating the SMAD family member 7 (Smad7) and NF-κB pathways (Yang et al., 2012), and suppressing c-Jun activation (Thiraworawong et al., 2014). The *L. salivarius* strain B37 produced a polysaccharide as an immunomodulatory factor of *IL-8* production in the gastric epithelium. In addition, the mixture of polysaccharides, lipids and proteins secreted by *L. salivarius* strain B60 was involved in mediating *IL-8* production (Panpetch et al., 2016). Moreover, animals receiving *Lactobacillus fermentum* P2, *Lactobacillus casei* L21, LR-JB3, or their combination had decreased *H. pylori*-specific IgA and IgM levels in the stomach, and IFN-γ and *IL-1*β levels in the serum (Lin et al., 2020).

#### 6.1.4. Communication between microorganisms: quorum sensing

The quorum sensing (QS) system is a molecular signaling mechanism for interbacterial communications to control their behavior, such as growth, virulence, and pathogenicity (Wu et al., 2021). The protein encoded by the S-ribosylhomocysteine lyase (*LuxS*) gene of *H. pylori* synthesizes autoinducer 2 (AI-2), which is a major molecule of QS (Forsyth and Cover, 2000). AI-2 has been reported to regulate *H. pylori* activity, including biofilm formation (Anderson et al., 2015) and motility (Rader et al., 2007). It has also been reported to reduce *CagA* expression and bacterial adhesion to attenuate the *H. pylori*-induced inflammatory response in gastric epithelial cells (Wen et al., 2021). In addition, AI-2 induces urease expression in *H. pylori* by downregulating the orphan response regulator HP1021, potentially enhancing acid acclimation when bacterial density increases (Yang et al., 2022).

QS is involved in the balance between the gut microbiota and the host. Many studies have gradually focused on QS-mediated interactions between different bacterial populations (Wu et al., 2021). Recently, Do et al. (2021a) found that LR-JB3 inhibited *LuxS* expression in *H. pylori*. An unknown bioactive signal secreted by LR-JB3 acts as an AI-2 signal antagonist, attenuating the effect of AI-2 and affecting the binding ability of *H. pylori* to AGS cells (Do et al., 2021a).

### 6.2. Prebiotics, postbiotics, synbiotics

Synbiotics are mixtures of living microorganisms (Swanson et al., 2020). Prebiotics are substrates selectively used by host health-promoting microorganisms (Gibson et al., 2017). Postbiotics are inanimate microorganisms and their components that confer a health benefit on the host (Salminen et al., 2021). The most widely documented dietary prebiotics in humans are the non-digestible oligosaccharides fructans and galactans (Gibson et al., 2017). Current postbiotic microorganism components include cell-free supernatants, bacterial lysates, cell wall fragments, exopolysaccharides, enzymes, and metabolites (SCFAs, vitamins, phenolic-derived metabolites, and aromatic amino acids) (Zolkiewicz et al., 2020).

A maternal-infant cohort study showed that dominant breastfeeding might prevent early *H. pylori* colonization (Shah et al., 2022). Human milk oligosaccharides (HMOs) unique to human milk were found to be prebiotic bifidus factors that promote colonization by *Bifidobacteria* members (Hill et al., 2021) and support cross-feeding among *Bifidobacteria* and other genera, such as butyrogenic *Anaerostipes caccae* (Chia et al., 2021). Postbiotic molecules, such as lactic acid (Arena et al., 2016) and bacteriocins (Kim et al., 2003), might have direct antimicrobial activity. However, postbiotics might also indirectly modulate the microbiota by carrying QS and quorum-quenching molecules (Grandclément et al., 2016). A meta-analysis of six randomized controlled trials suggested that synbiotics might improve *H. pylori* eradication rates and reduce adverse effects (Ustundag et al., 2017).

### 6.3. Microbiota transplantation

Fecal microbiota transplantation (FMT) has been used to effectively restore the GI microbiota to treat GI diseases, such as *Clostridium difficile* infection and inflammatory bowel disease (Allegretti et al., 2019). Washing microflora transfer (WMT) is a modified FMT method that uses washed preparations. WMT application *via* the stomach, jejunum, or right hemicolon delivery routes caused an overall *H. pylori* eradication of 40.6% in a cohort of 32 *H. pylori*-infected patients, which was significantly associated with an increased pre-WMT PG ratio. It should be noted that the relationship between the curative effect, sex, and delivery route (upper, middle, and lower GI tract) requires further investigation (Ye et al., 2020).

In healthy adults, the bacterial community differs not only in individuals but also in different GI regions of the same individual. Therefore, the fecal microbiome is not representative of the mucosal microbiome (Vasapolli et al., 2019). A recent study found that GF mice fed gastric mucosal tissue and juice from patients with IM or GC were colonized by specific human gastric microorganisms. Moreover, they recapitulated the major histopathological features of premalignant changes (Kwon et al., 2022). The total number of ILC2s in the stomach was lower in GF mice than in SPF mice. However, ILC2 numbers and IL-5 levels were elevated after stomach microbiota transfer by gavage of stomach contents and mucosal scraping from SPF mice to GF mice, correlating with the increased relative abundance of Bacteroidales family S24-7 (Satoh-Takayama et al., 2020). Although research on gastric microbiota transplantation (GMT) in *H. pylori* eradication is lacking, it appears to have broad prospects.

## 7. Expectation

**FIGURE 2:**
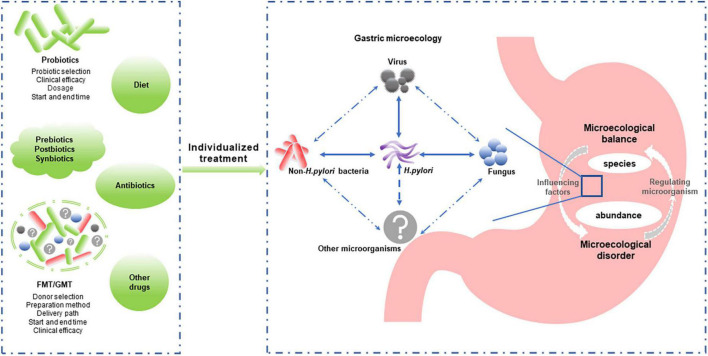
Individualized reconstruction of the healthy gastric microbiota is a promising strategy for managing microecology dysbiosis-associated gastric diseases. Gastric microbiota composition and abundance and the interaction between gastric microbiomes (including *Helicobacter pylori* and non-*H. pylori* bacteria, fungi, and viruses) play important roles in gastric microecological homeostasis. Modulating the microbiota (probiotics, prebiotics, postbiotics, synbiotics, and FMT/GMT) is expected to improve and restore the gastric microflora balance. However, individualized treatment options, such as the bacteria type or donor selection, delivery path, and start and end times, require further study. FMT, fecal microbiota transplantation; GMT, gastric microbiota transplantation.

While the importance of non-*H. pylori* bacteria in gastric diseases has been highlighted by in-depth gastric microecology studies, the role of *H. pylori* cannot be ignored. Some scholars believe that the potential protective effects of *H. pylori* for some diseases, such as inflammatory bowel disease (Engler et al., 2015), need to be taken seriously, and *H. pylori* should even be considered a commensal organism, not just an opportunistic pathogen (Reshetnyak et al., 2021). Similarly, many *Lactobacillus* species used as probiotics play a role in preventing pathogen infection, reducing inflammation, and modulating the microbiota. However, *Lactobacillus* was also able to induce inflammatory damage to epithelial cells and was associated with GC (Vinasco et al., 2019). Whether the balance between its beneficial and detrimental effects is related to specific bacterial species or abundance is worthy of further study. Considering the balance of the gastric microecology (e.g., bacteria, fungi, and viruses) rather than the role of specific bacteria may provide us with new approaches for preventing and treating gastric diseases (Figure 2).

Regulating gastric microecology might play an important role in *H. pylori* eradication. Oral microbe administration always leads to a substantial loss of viability due to the highly acidic environment of the stomach (Li S. et al., 2020). Host factors influencing probiotic colonization and efficacy include diet, age, antibiotic use, underlying medical conditions, and baseline microbiome composition and function (Suez et al., 2020). In addition, studies have found that probiotic colonization resistance is partly due to the indigenous gut microbiome (Zmora et al., 2018). Antibiotic therapy in healthy individuals can partially overcome probiotic colonization resistance due to the homeostatic microbiome, improving probiotic colonization in the depleted gut mucosal layer (Suez et al., 2018). Therefore, the clinical efficacy of probiotics against *H. pylori* requires larger samples and more extended observation, and individualized treatment plans need to be further developed (Figure 2).

Furthermore, microbiome transplantation induced a rapid and nearly complete reconstitution of the gut microbiome after antibiotic treatment. Therefore, it appears to provide rapid postantibiotic protection during the nadir period of the intestinal mucosal microbiome compared to structurally single probiotics (Suez et al., 2018). GMT is a promising strategy for restoring normal gastric microbiota that requires further investigation (Figure 2).

## Author contributions

LZ drafted the preliminary manuscript. MZ and XF refined and approved the final manuscript. All authors contributed to the article and approved the submitted version.
